# Inhibition of NLRP3 inflammasome contributes to paclitaxel efficacy in triple negative breast cancer treatment

**DOI:** 10.1038/s41598-024-75805-3

**Published:** 2024-10-21

**Authors:** Liliana-Roxana Balahura Stămat, Sorina Dinescu

**Affiliations:** 1 Department of Biochemistry and Molecular Biology, Faculty of Biology, University of Bucharest, Bucharest, 050095 Romania; 2 Research Institute of the University of Bucharest, Bucharest, 050663 Romania

**Keywords:** TNBC, NLRP3 inflammasome, Paclitaxel, MCC950, siRNA, Cancer, Cell biology, Molecular biology

## Abstract

Chronic inflammation and NLRP3 inflammasome activation are among the determining factors of breast malignancies. Paclitaxel (PTX) is a drug used in breast cancer treatment which sustains prolonged inflammation, reducing the effectiveness of chemotherapy. Considering the impact of inflammatory processes in cancer progression, there is a strong concern to develop therapeutic strategy targeting NLRP3 inflammasome for triple-negative breast cancer (TNBC) treatment. Therefore, the aim of this study was to evaluate the potential of PTX and NLRP3 inflammasome modulation to counterbalance TNBC by inducing programmed cell death and inhibiting the activity of pro-inflammatory cytokines. The obtained results suggested the strong interaction between NLRP3 inflammasome and TNBC and revealed that pharmacological inhibition, using NLRP3-specific inhibitor MCC950, and genetic silencing of NLRP3 inflammasome using specific small interfering RNA, reduced inflammatory responses and facilitated PTX-determined tumor cell death. Thus, NLRP3 inflammasome manipulation in combination with anti-tumor drugs opens up new therapeutic perspectives for TNBC therapy.

## Introduction

Triple-negative breast cancer (TNBC) is an aggressive and heterogeneous pathology whose treatment remains challenging. Over the years, efforts have been made to elucidate the molecular landscape of TNBC and the results revealed the complex interaction between breast malignancy and chronic inflammation^1^. Malignant transformation of the inflamed breast tissue is sustained by pro-inflammatory microenvironment and the activation of pro-inflammatory signaling pathways, such as nuclear factor kappa-light-chain-enhancer of activated B cells (NF-kB)^2^. Inflammation-related components released by tumor cells influence the majority of signaling pathways and processes involved in breast carcinogenesis, thus inflammation is considered a major risk factor for TNBC development and progression^3^. In TNBC context, the presence of pro-inflammatory cytokines, such as tumor necrosis factor α (TNFα) and interleukin-1β (IL-1β) is associated with tumor growth and highly metastatic potential^4^. Also, several studies suggested that the control of inflammation is a key factor of an efficient anti-tumor strategy^5^.

In particular, special interest has been given to understand the NOD-like receptor family (NLR), pyrin domain containing 3 (NLRP3) inflammasome activation mechanism and its consequences on tumor initiation, progression and metastasis. The canonical activation of NLRP3 inflammasome is based on two signals: priming and activation. The priming signal can be achieved by the detection of damage-associated molecular pattern molecules (DAMPs) or pathogen-associated molecular patterns (PAMPs) by Toll-like receptors (TLR), which will stimulate NF-kB activation and transcription of NLRP3 and pro-inflammatory cytokines, while the activation signal determines the oligomerization of NLRP3 inflammasome in the presence of apoptosis-associated speck-like protein containing a caspase recruitment domain (CARD) (ASC) and pro-caspase-1^6^. The NLRP3 sensor contains a leucine-rich repeat (LRR) domain at the carboxyl terminal end, a pyrin domain (PYD) at the amino terminal end and a nucleotide-binding domain (NACHT) in the center domain. The detection of specific stimuli by the receptors determines PYD-PYD interaction between NLRP3 and ASC and CARD-CARD interaction between ASC and pro-caspase-1 which promote NLRP3 complex formation^7^. Mature caspase-1 cleaves pro-IL-1β and pro-IL-18 to produce corresponding mature cytokines and pore-forming gasdermin D (GSDMD) in order to promote the caspase-1-dependent cell death, named pyroptosis^8^.

TNBC microenvironment can promote carcinogenesis through NLRP3 inflammasome activation^9^. NLRP3 mechanism is involved in diverse cellular processes, such as cell growth, programmed cell death, inflammatory and immune reactions^8^, allowing breast tumor cells to escape immunosurveillance and promoting inflammation-associated tumor progression^10^. Additionally, among all the previously studied markers in TNBC progression, NLRP3 inflammasome was the most studied and with the most consistent results that can make it a reproducible target for TNBC therapy^11^. NLRP3 inflammasome activation determines caspase-1 maturation, GSDMD cleavage and formation of GSDMD-derived pores, stimulating pyroptosis initiation. Inflammatory processes are stimulated by the presence of GSDMD-derived pores, and implicitly pyroptosis, due to calcium/sodium ions influx and potassium ions efflux, as cells become swollen, lysed, nucleated and fragmented^12^.

A special interest in the oncological field is also given to paclitaxel (PTX), a tricyclic diterpenoid compound isolated from the bark of a medicinal plant named *Taxus brevifolia*, which has been used as anti-cancer agent for breast cancer treatment since 1994^13^. Moreover, advanced studies revealed the mechanism of action of PTX and outlined the ability of this anti-tumor agent to interact with microtubule activity^14^. PTX can attach to the structure of microtubules, causing their stabilization and collapse, subsequently blocking the cell cycle in mitosis and initiating the caspase- or cathepsin-dependent cell death programs, pyroptosis or autophagy^15^. Classic anti-tumor therapies are ineffective in TNBC pathology considering its aggressiveness and molecular characteristics, such as the absence of hormone receptors and invasive potential of tumor cells. Therefore, the need to develop targeted therapies has increased significantly and a promising candidate has been proved to be the NLRP3 inflammasome due to simultaneous targeting of caspase-1, IL-1β and IL-18^16^.

Over the years numerous studies have focused on targeting the NLRP3 inflammasome pathway using both NLRP3 inflammasome synthetic inhibitors (e.g. MCC950) or non-coding RNA molecules that intervene at the molecular level^17^. The activation mechanism of the inflammasome is stimulated by multiple factors, including adenosine triphosphate (ATP), lipopolysaccharides (LPS) or uric acid crystals^18^. ATP can trigger the activation of NLRP3 inflammasome by binding to the P2X7R purinergic receptor and opening the ion channel for ATP and potassium efflux^19^. LPS is the major constituent Gram-negative bacteria membrane, which is detected by TLR4 and activates the NF-kB signaling cascade, the secretion of pro-inflammatory cytokines and the innate immune system^20^. On the other hand, MCC950 is considered the most selective and effective inhibitor of the NLRP3 inflammasome, having no inhibitory effect on the activation of the other types of inflammasomes^21^. MCC950 abrogates ASC oligomerization and IL-1β and IL-18 maturation, blocks ATP hydrolysis and inflammasome formation by binding to NLRP3 sensor^22^. Therefore, MCC950 is considered the most potent and specific inhibitor of the NLRP3 inflammasome, being used for modulation and evaluation of NLRP3 functions^23^.

In this study, we evaluated the combined effects of MCC950 and PTX on MDA-MB-231 cells, compared with MCF-12 A cells, considering that MCC950 is the most specific and efficient inhibitor of NLRP3 which alter the NLRP3 inflammasome conformation by binding the NACHT domain and implicitly affecting the associated inflammatory responses^24^ and the PTX`s cytotoxic effects on breast tumor cells, being a first-line chemotherapy drug used in clinical practices^25^.

RNA interference mechanism allows the silencing of a corresponding messenger RNA (mRNA) using a small interfering RNA (siRNA) molecule^26^. SiRNAs are double stranded RNA molecules which mediate silencing of target genes by guiding sequence dependent slicing of their target mRNAs^27^. SiRNA molecules are the functional intermediates of gene silencing which is a process that occurs at the post-transcriptional level and consists of two steps: siRNA production and mRNA degradation. The siRNA interacts with Argonaute-2 protein and is incorporated into the RNA-induced silencing complex (RISC), resulting in duplex breakage and degradation of the lagging strand. The leading strand, which is complementary to the target mRNA, guides the RISC complex to the target mRNA molecule^28^. Therefore, siRNA-based gene silencing strategies may serve as an effective targeted therapy for NLRP3 inflammasome in order to reduce inflammation and to facilitate the anti-tumor drugs effects.

Consequently, the aim of the current study was to contribute to a better understanding of NLRP3 inflammasome signaling in TNBC pathogenesis and to observe the capacity of PTX to influence NLRP3 inflammasome activation, maturation of caspase-1 and secretion of IL-1β under pharmacological inhibition or genetic silencing of NLRP3 inflammasome, in order to provide a possible effective anti-tumor strategy.

## Results

### PTX effect on NLRP3 inflammasome activation

#### NLRP3 inflammasome-associated gene expression levels

**Fig. 1:**
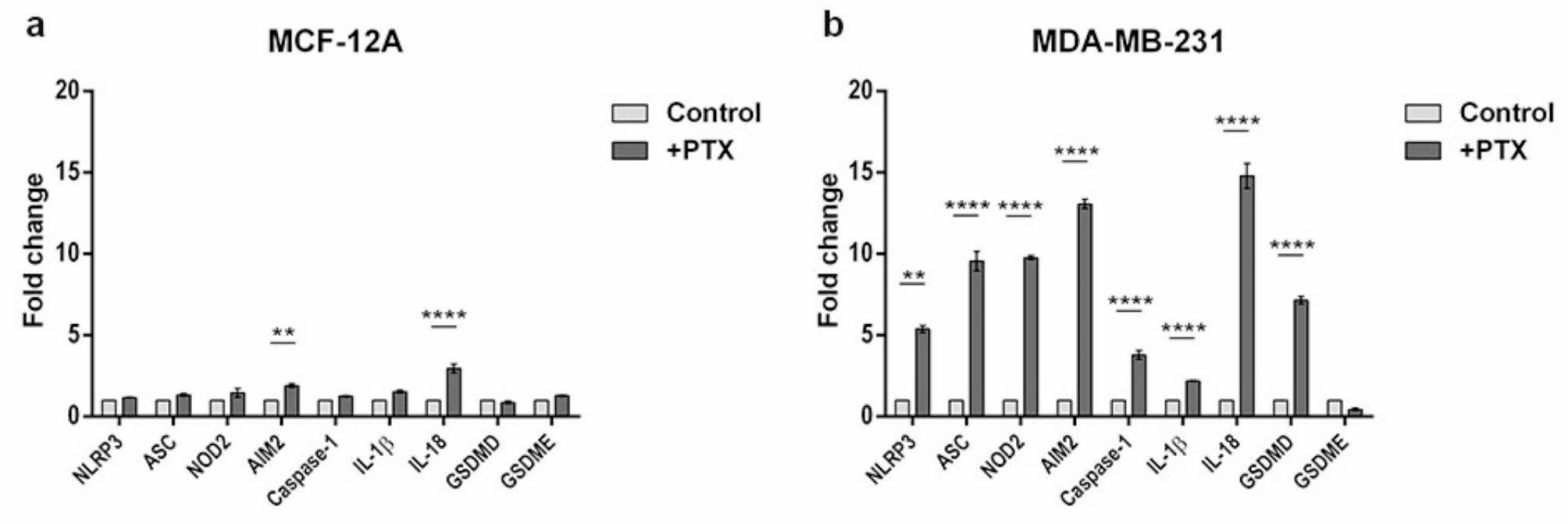
Differential NLRP3 inflammasome - associated gene expression levels. The expression of genes involved in breast carcinogenesis and NLRP3 inflammasome activation before and after (a) MCF-12A and (b) MDA-MB-231 cells` treatment with 100 nM PTX for 24 h. Statistical significance: ** *p* < 0.01, *** *p* < 0.001 and **** *p* < 0.0001 (*n* = 3, biological replicates).

The capacity of PTX to mediate the activation of inflammasome was examined at the gene level. The differentially expressed genes between normal and TNBC cells after PTX treatment were screened using qRT-PCR (Fig. 1). The obtained data indicated that PTX increased the expression of genes associated with inflammasome assembly in MDA-MB-231 cells, such as NLRP3 (*p* < 0.01), ASC (*p* < 0.0001), NOD2 (*p* < 0.0001), AIM2 (*p* < 0.0001) and caspase-1 (*p* < 0.0001). The up-regulation of these genes, especially NLRP3 and AIM2, suggest the activation of inflammasome mechanism by recruiting caspase-1 via ASC. Pyroptosis initiation was also suggested by the statistically significant up-regulated profile of pro-inflammatory IL-1β (*p* < 0.0001) and IL-18 (*p* < 0.0001) and pore-forming GSDMD (*p* < 0.0001), while no relevant differences were noted in GSMDE expression as following PTX treatment, compared to control. GSDMD is a co-executor of pyroptosis, which facilitates the release of cell contents, including pro-inflammatory cytokines IL-1β and IL-18^29^. On the other hand, GSDME expression in MDA-MB-231 tumor cells exposed to PTX was insignificantly modified, similar results being obtained by Hou et al., 2020^30^. In contrast, PTX treatment determined statistically significant increased profiles only for AIM2 (*p* < 0.01) and IL-18 (*p* < 0.0001) in MCF-12A cells, suggesting the initiation of an inflammatory reaction as a consequence of chemotherapy. However, the expression levels of the other NLRP3 inflammasome-associated components after PTX treatment were slightly modified compared to untreated control. These data indicate that PTX induces NLRP3 inflammasome mechanism in MDA-MB-231 cells, while on MCF-12 A cells PTX exhibited limited effect.

#### PTX effect on caspase-1 and IL-1β activity

**Fig. 2:**
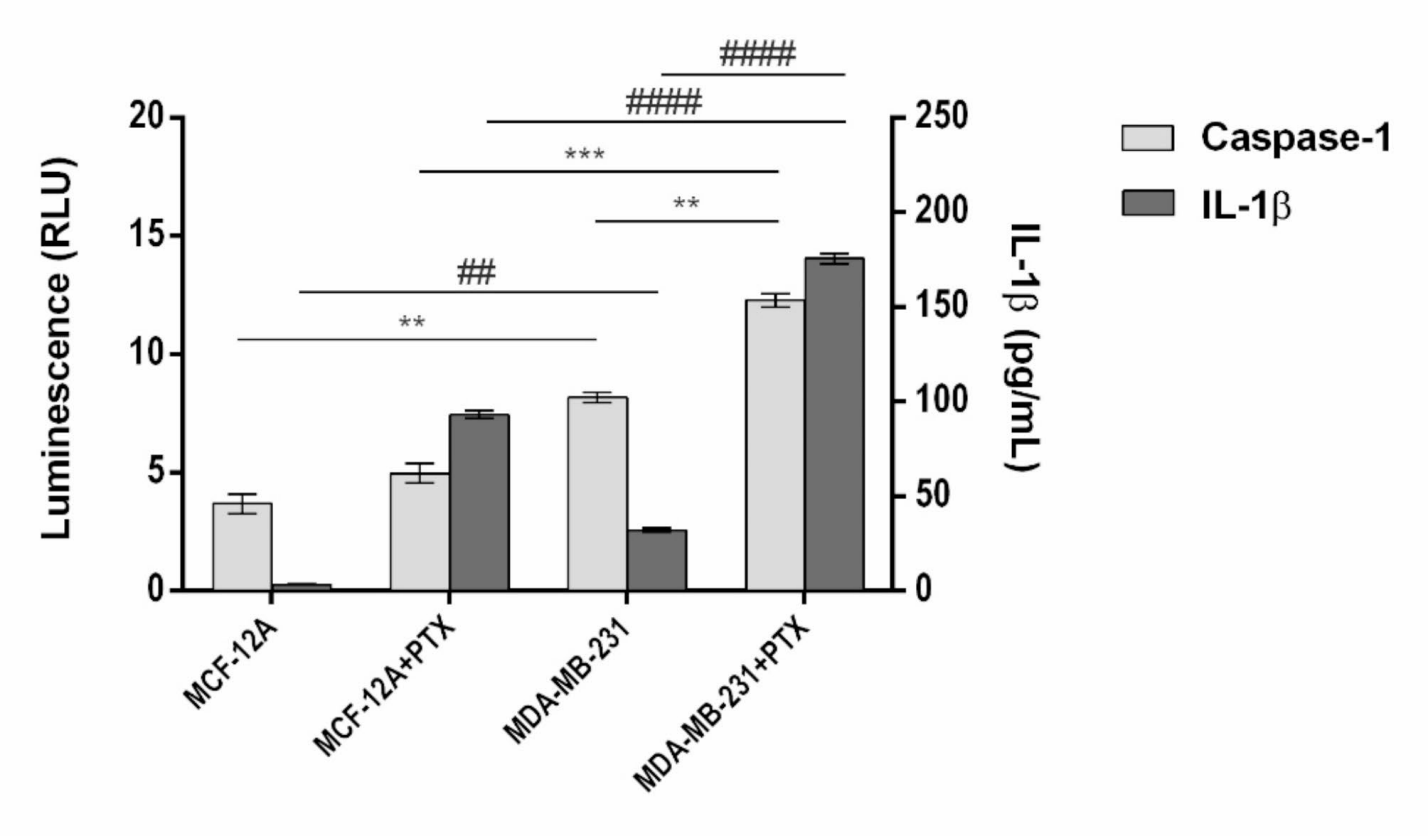
Multiplexing the Lumit Human IL-1β Immunoassay with the Caspase-Glo 1 Inflammasome Assay. MCF-12A and MDA-MB-231 cells were treated with 100 nM PTX for 24 h. Statistical significance: “*” symbol was used for statistical comparisons between the levels of caspase-1 secreted in each experimental condition; “#” symbol was used for statistical comparisons between the levels of IL-1β secreted in each experimental condition; ** *p* < 0.01, *** *p* < 0.001, ## *p* < 0.01 and #### *p* < 0.0001 (*n* = 3, biological replicates).

PTX capacity to induce the activation of caspase-1 and the secretion of IL-1β in TNBC cells compared to normal cells was determined using two bioluminescent methods (Fig. 2). Slightly ascending but no statistically significant profiles of caspase-1 suggested the cytotoxic effect of PTX on MCF-12A cells. The property of tumor cells, especially this type of aggressive breast cancer cells, to produce pro-inflammatory mediators was highlighted by the statistically increased profile of caspase-1 in untreated MDA-MB-231 cells compared to MCF-12A cells (*p* < 0.01). On the other hand, the administration of PTX to tumor cells caused a significant increase in caspase-1 activity compared to untreated tumor cells (*p* < 0.01). Overall, the PTX administration caused the acceleration of caspase-1 production in MDA-MB-231 compared to MCF-12A (*p* < 0.001).

TNBC-related inflammation is also confirmed by the statistically increased release of IL-1β by tumor cells compared to normal cells (*p* < 0.01). PTX treatment robustly induced the secretion of pro-inflammatory IL-1β in MDA-MB-231 cells compared to control MCF-12A cells (*p* < 0.01). Changes in cytokine`s levels were statistically increased after the PTX treatment of breast cells (*p* < 0.0001), suggesting the influence of this anti-tumor drug to interfere with NLRP3 inflammasome activation and pro-inflammatory mediators secretion in TNBC MDA-MB-231 cells.

#### NLRP3 inflammasome-associated protein expression levels

**Fig. 3:**
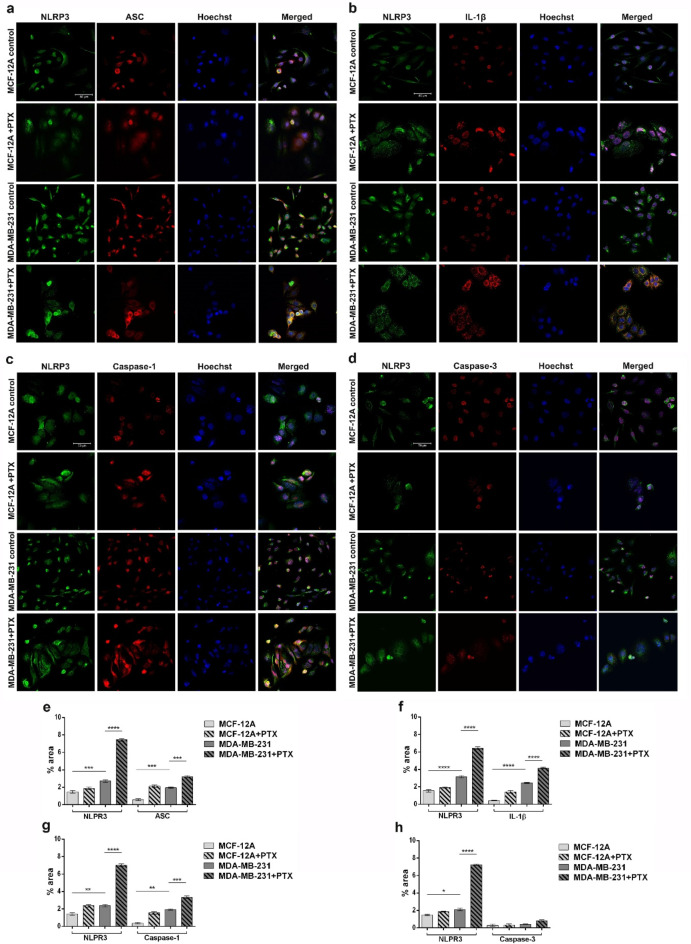
Expression of NLRP3, ASC, IL-1β, caspase-1 and caspase-3 protein in normal and tumor cells treated with PTX. Confocal immunofluorescence microscopy images revealing (a) NLRP3 and ASC, (b) NLRP3 and IL-1β, (c) NLRP3 and caspase-1, (d) NLRP3 and caspase-3 protein expression in MCF-12A and MDA-MB-231 cells before and after treatment with 100 nM PTX for 24 h (scale bar 50 μm). NLRP3 is shown green (AF488), ASC, IL-1β, caspase-1 and caspase-3 in red (AF546) and cell nuclei in blue (Hoechst 33342). (e, f, g, h) Quantitative measurement of fluorescence intensity was performed with ImageJ software. Statistical significance: * *p* < 0.05, ** *p* < 0.01, *** *p* < 0.001 and **** *p* < 0.0001.

The protein expression of NLRP3 inflammasome components was evaluated by immunofluorescence coupled with confocal microscopy and confirmed the gene expression results. Low NLRP3 and ASC expression levels were observed in untreated MCF-12A and slightly increased after PTX addition. Additionally, the protein expression of NLRP3 and ASC was noticeably higher in tumor cells compared to normal cells and substantially increased after anti-tumor drug treatment (Fig. 3a). After 24 h of incubation with PTX, the expression of IL-1β was moderately detected in MCF-12A cells, while in MDA-MB-231 was noticeably higher as compared to untreated cells (Fig. 3b). Along with the increase of NLRP3, ASC and IL-1β expression levels, an increasing caspase-1 expression profile was obtained for PTX-treated tumor cells compared untreated cells. Meanwhile, caspase-1 was poorly expressed on control MCF-12A cells before and after treatment (Fig. 3c). Regarding the levels of caspase-3 no significant variation was observed following PTX administration neither in the case of normal cells neither tumor cells (Fig. 3d), suggesting the absence of caspase-3/GSDME signaling pathway. Fluorescent labeling allowed the qualitative analysis of inflammasome components` expression before and after PTX treatment. NLRP3 inflammasome and pyroptosis activation in PTX-treated MDA-MB-231 cells were suggested by the statistically increased levels of NLRP3 (*p* < 0.0001), ASC (*p* < 0.001), IL-1β (*p* < 0.0001) and caspase-1 (*p* < 0.001) as compared to untreated MDA-MB-231 cells, while caspase-3 levels were insignificantly modified (Fig. 3e, f, g, h).

### The effects of LPS and/or ATP stimulation and MCC950 inhibition on PTX activity

#### NLRP3-associated genes expression after pharmacological modulation of NLRP3

**Fig. 4:**
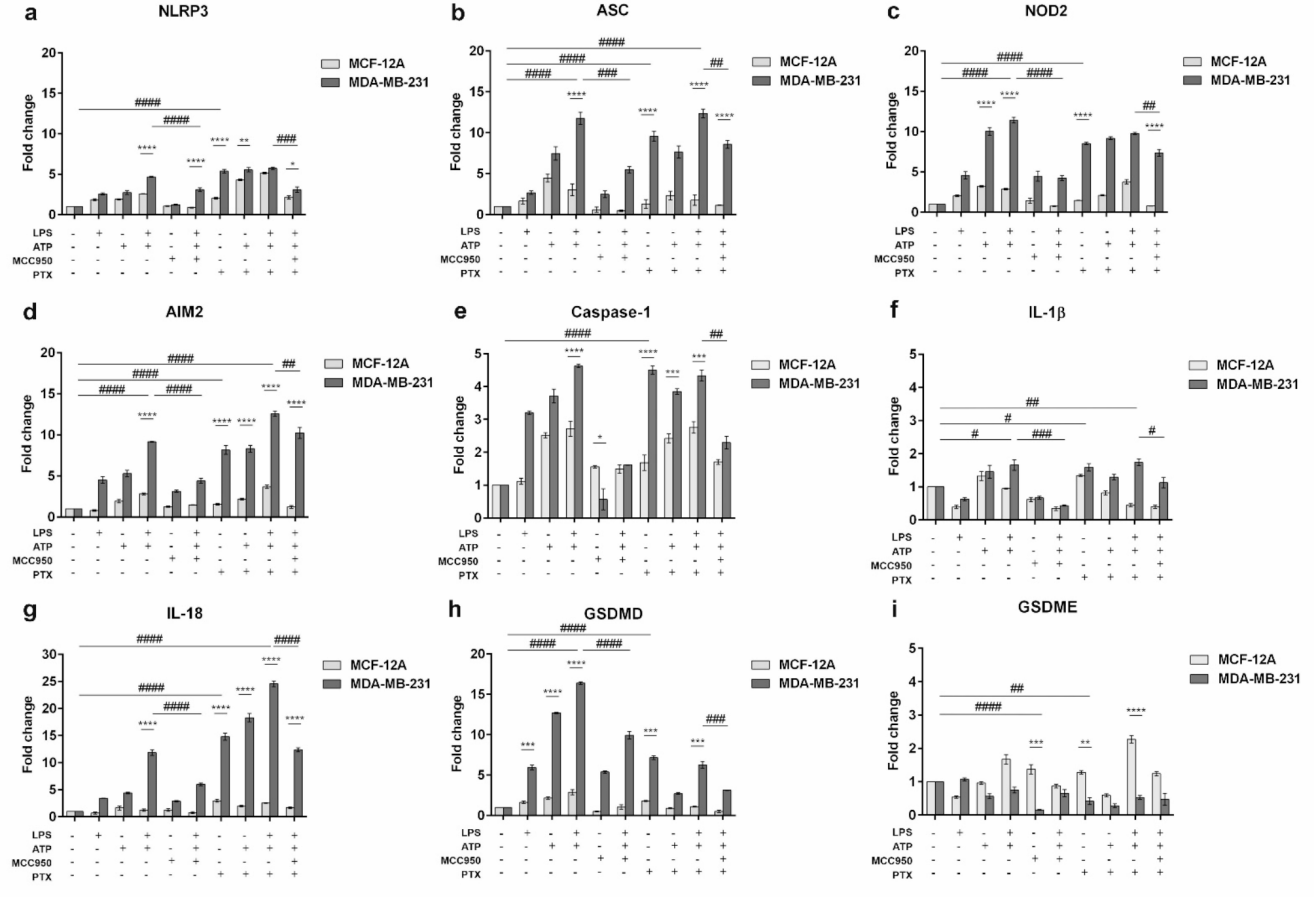
The effects of NLRP3 pharmacological modulation on gene expression. Differential expression in MCF-12A and MDA-MB-231 cells of (a) NLRP3, (b) ASC, (c) AIM2, (d) NOD2, (e) IL-18, (f) IL-1β, (g) caspase-1, (h) GSDMD and (i) GSDME genes. MCF-12A and MDA-MB-231 cells were primed with 1 µg/mL LPS for 4 h followed by treatment with 5 mM ATP for an additional 1 h and then exposed to 10 µM MCC950 and/or 100 nM PTX for 24 h. Statistical significance: “*” symbol was used for statistical comparisons between cell lines; “#” symbol was used for statistical comparisons between experimental condition; * *p* < 0.05, ** *p* < 0.01, *** *p* < 0.001, **** *p* < 0.0001, # *p* < 0.05, ## *p* < 0.01, ### *p* < 0.001 and #### *p* < 0.0001 (*n* = 3, biological replicates).

Gene expression analysis suggested the potential of NLRP3 inflammasome modulation to counterbalance breast cancer cells growth by activating the immunogenic cell death (pyroptosis) and inhibiting the activity of pro-inflammatory cytokines, IL-1β and IL-18 (Fig. 4). Stimulation of tumor cells with LPS followed by ATP addition determined an increase in NLRP3-associated genes levels, which were subsequently decreased following MCC950-based inhibition. Moreover, PTX treatment enhanced LPS and ATP effect on NLRP3 inflammasome and pyroptosis activation.

LPS and ATP stimulation determined the inflammasome assembly in TNBC cells as revealed by statistically increased expression of NLRP3, ASC, NOD2 and AIM2 (*p* < 0.0001), while pyroptosis initiation was suggested by the increase of caspase-1 (*p* < 0.0001), IL-1β, IL-18 (*p* < 0.0001) and GSDMD (*p* < 0.0001). Decreased GSDME expression in LPS and ATP-treated tumor cells confirmed the existing data illustrating the down-regulation of GSDME in breast cancer conditions^31^. After NLRP3-specific inhibitor MCC950 was added, the levels of NLRP3, ASC, NOD2 and AIM2 statistically decreased (*p* < 0.001 and *p* < 0.0001) upon stimulation with LPS and ATP of tumor cells. Caspase-1-dependent cell death was also constrained by the presence of MCC950 as indicated by the low expression of pyroptosis` effectors.

On the other hand, the potential of PTX to induce the inflammasome and pyroptosis mechanisms were also studied. PTX treatment significantly increased inflammasome activation, as indicated by significantly increased NLRP3, ASC, NOD2 and AIM2 (*p* < 0.0001) expression in MDA-MB-231 tumor cells compared to control cells. PTX administration potentiated pyroptosis by increasing the expression of pro-inflammatory mediators and programmed cell death effectors, caspase-1 (*p* < 0.0001) and GSDMD (*p* < 0.001). Although simple administration of PTX enhanced NLRP3 inflammasome mechanism, we studied whether PTX could enhance LPS and ATP-induced pyroptosis. The results indicated that PTX promoted NLRP3 inflammasome activation and pyroptosis induced by LPS and ATP pre-treatment of TNBC cells.

PTX treatment increased the expression of inflammasome components and pyroptosis effectors upon stimulation with LPS and ATP, whereas inhibition of NLRP3 complex with MCC950 attenuated the response to PTX of MDA-MB-231 cells. The obtained results indicate that MCC950 inhibited the activation of inflammasome by decreasing the levels of NLRP3 (*p* < 0.001), ASC (*p* < 0.01), NOD2 (*p* < 0.01), AIM2 (*p* < 0.01), caspase-1 (*p* < 0.01), IL-1β (*p* < 0.05), IL-18 (*p* < 0.0001) and GSDMD (*p* < 0.001) induced by the stimulators (LPS, ATP and PTX). Therefore, the inhibition of the NLRP3 and implicitly the decrease of the pro-inflammatory markers can reduce the side effects of the PTX treatment, facilitating the anti-tumor therapy.

#### Caspase-1 and IL-1β variation after pharmacological modulation of NLRP3

**Fig. 5:**
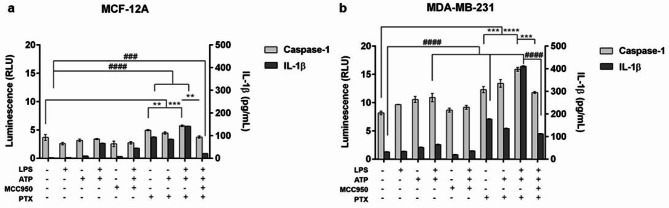
Multiplexing the Lumit Human IL-1β Immunoassay with the Caspase-Glo 1 Inflammasome Assay. (a) MCF-12A and (b) MDA-MB-231 cells were primed with 1 µg/mL LPS for 4 h followed by treatment with 5 mM ATP for an additional 1 h and then exposed to 10 µM MCC950 and/or 100 nM PTX for 24 h. Statistical significance: “*” symbol was used for statistical comparisons between the levels of caspase-1 secreted in each experimental condition; “#” symbol was used for statistical comparisons between the levels of IL-1β secreted in each experimental condition; ** *p* < 0.01, *** *p* < 0.001, **** *p* < 0.0001, ### *p* < 0.001 and #### *p* < 0.0001 (*n* = 3, biological replicates).

Thereafter, the effects of inflammasome modulation on pyroptosis were studied as determined by the release of caspase-1 and IL-1β in culture supernatant (Fig. 5). Stimulation with LPS and/or ATP alone did not cause a significant cell death neither in normal nor in tumor cells. Treatment with PTX strongly supported caspase-1 activation (*p* < 0.001) and IL-1β release (*p* < 0.0001) in MDA-MB-231 cells compared to untreated cells, but it also affected the behavior of normal cells. In addition, LPS, ATP and PTX stimulation determined dynamic activation of caspase-1 and IL-1β in MCF-12A cells (*p* < 0.001 and *p* < 0.0001 respectively) and MDA-MB-231 cells (*p* < 0.0001) compared to appropriate control. On the other hand, the inhibition of NLRP3 inflammasome using MCC950 reduced LPS, ATP, PTX-mediated caspase-1 and IL-1β release, both in normal cells (*p* < 0.01 and *p* < 0.001, respectively) and tumor cells (*p* < 0.001 and *p* < 0.0001, respectively).

### NLRP3 siRNA-mediated knock-down

#### NLRP3 gene levels variation after PTX treatment and NLRP3 knock-down

**Fig. 6:**
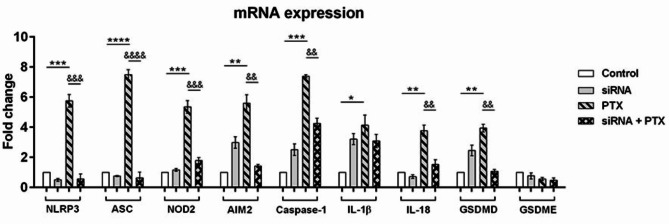
Differential expression in genes involved in breast carcinogenesis and NLRP3 inflammasome activation. MDA-MB-231 cells were transfected with NLRP3 siRNA for 72 h followed by incubation with 100 nM PTX for an additional 24 h. Statistical significance: “*” symbol was used for statistical comparisons between control and 100 nM PTX treatment; “#” symbol was used for statistical comparisons between 100 nM PTX treatment and NLRP3 siRNA transfection combined with 100 nM PTX; * *p* < 0.05, ** *p* < 0.01, *** *p* < 0.001, **** *p* < 0.0001, && *p* < 0.01, &&& *p* < 0.001 and &&&& *p* < 0.0001 (*n* = 3, biological replicates).

The results obtained by pharmacological modulation of NLRP3 inflammasome were confirmed using siRNA-mediated knock-down of NLRP3 (Fig. 6). The silencing of the NLPR3 gene decreased ASC and NOD2 expression profile, highlighting their function as molecular platforms for inflammasome assembly. Considering NLRP3 and ASC decreased gene expression, the up-regulation of AIM2, caspase-1, IL-1β, IL-18 and GSDMD suggest the initiation of a pro-inflammatory signaling pathway. MDA-MB-231 cells were exposed to PTX before and after NLRP3 knock-down and the results indicated up-regulation of inflammasome components, such as NLRP3 (*p* < 0.001), ASC (*p* < 0.0001), NOD2 (*p* < 0.001) and pyroptosis, such as caspase-1 (*p* < 0.001), IL-1β (*p* < 0.05), IL-18 (*p* < 0.01), GSDMD (*p* < 0.01) in PTX-treated tumor cells. PTX-treated tumor cells transfected with NLRP3 siRNA were unable to up-regulate NLRP3 and ASC expression. On the other hand, NLRP3 knock-down followed by PTX treatment altered the mechanism of pyroptosis as indicated by the statistically decreased expression of caspase-1 (*p* < 0.01), IL-18 (*p* < 0.01), GSDMD (*p* < 0.01) compared to PTX treatment only.

#### Silencing of NLRP3 constrains caspase-1 and IL-1β production

**Fig. 7:**
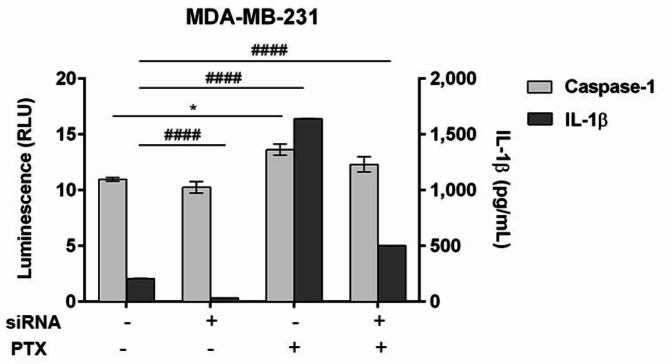
Multiplexing the Lumit Human IL-1β Immunoassay with the Caspase-Glo 1 Inflammasome Assay. MDA-MB-231 cells were transfected with NLRP3 siRNA for 72 h followed by incubation with 100 nM PTX for an additional 24 h. Statistical significance: “*” symbol was used for statistical comparisons between the levels of caspase-1 secreted in each experimental condition; “#” symbol was used for statistical comparisons between the levels of IL-1β secreted in each experimental condition; * *p* < 0.05 and #### *p* < 0.0001 (*n* = 3, biological replicates).

PTX-induced caspase-1 activation and IL-1β secretion was reduced in NLRP3 knock-down MDA-MB-231 cells (Fig. 7). Caspase-1 activity slightly decreased after NLRP3 gene silencing suggesting the reduction of the inflammatory response. However, PTX treatment determined a statistically significant increase of caspase-1 production in untreated cells (*p* < 0.05) and slightly increased but insignificantly in NLRP3 knock-down cells compared to control. SiRNA-mediated knock-down of NLRP3 statistically decreased IL-1β secretion in MDA-MB-231 cells compared to control (*p* < 0.0001). As expected, PTX statistically increased IL-1β production in MDA-MB-231 cells (*p* < 0.0001), but it can be observed that NLRP3 knock-down constrained IL-1β activity.

#### Inflammasome-associated protein expression levels in NLRP3 knock-down cells

**Fig. 8:**
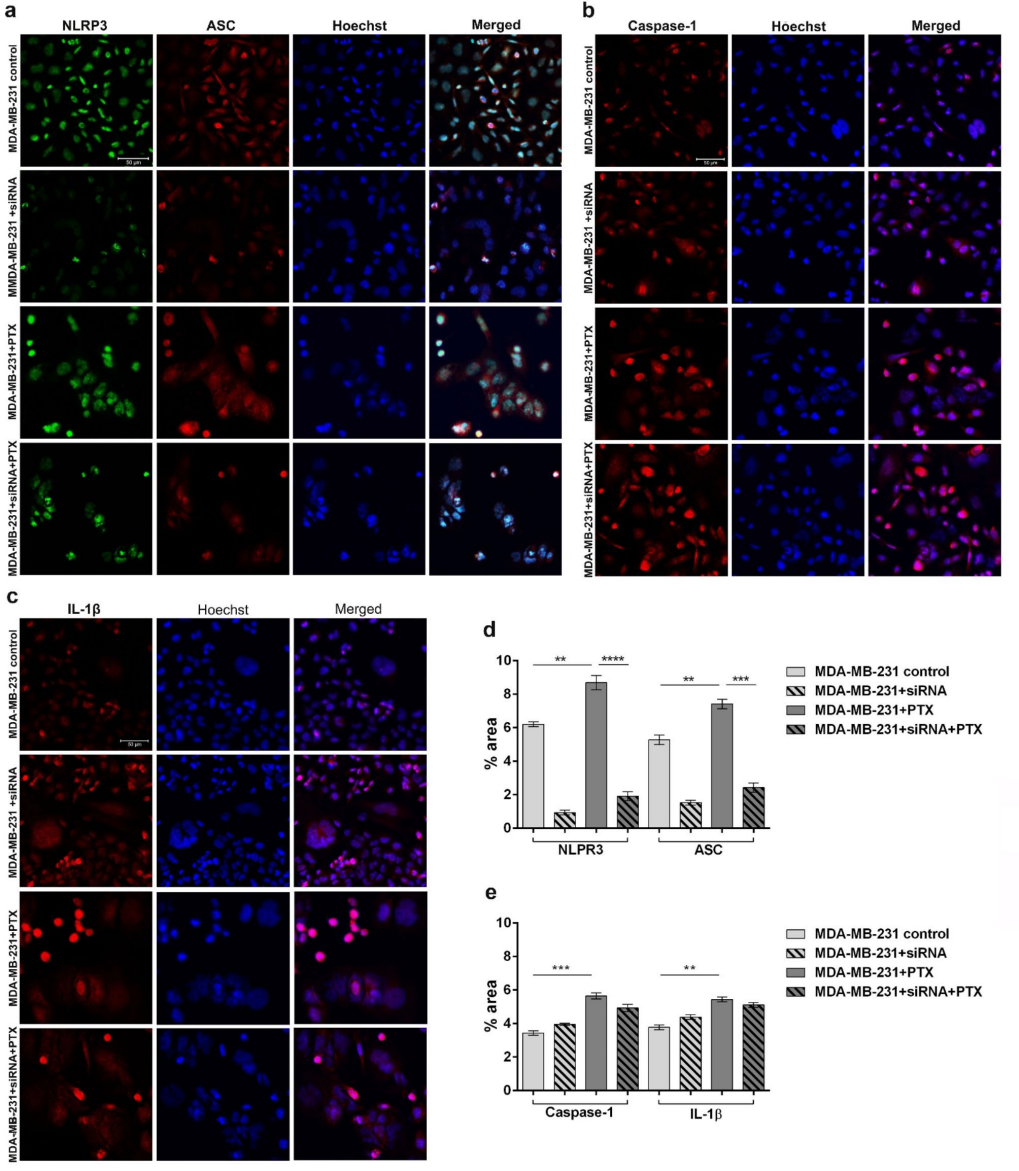
NLRP3 inflammasome – related protein expression in NLRP3 knock-down cells before and after exposure to 100 nM PTX. Confocal immunofluorescence microscopy images revealing (a) NLRP3, ASC, (b) caspase-1 and (c) IL-1β protein expression in MDA-MB-231 cells before and after NLRP3 siRNA-mediated knock-down and 100 nM PTX treatment (scale bar 50 μm). NLRP3 is shown green (AF488), while ASC, IL-1β and caspase-1 in red (AF546) and cell nuclei in blue (Hoechst 33342). (D, E) Quantitative measurement of fluorescence intensity was performed with ImageJ software. Statistical significance: ** *p* < 0.01, *** *p* < 0.001 and **** *p* < 0.0001.

Activation of NLRP3 inflammasome is dependent on the dynamic assembly between NLRP3 and ASC, which determined the maturation of caspase-1 and secretion of IL-1β. Confocal microscopy coupled with immunofluorescence confirmed the efficiency of NLRP3 knock-down as indicated by the reduced expression of NLRP3 in untreated and PTX-treated MDA-MB-231 even if the taxol presence determined a slight increase in the expression of NLRP3 compared to control (Fig. 8a). Moreover, ASC expression was notably inhibited in TNBC cells treated with NLRP3 siRNA, suggesting the crucial role of ASC oligomerization in the assembly and activation of NLRP3 inflammasome (Fig. 8b). Similar, ASC expression in NLRP3 knock-down cells slightly increased after PTX treatment, highlighting the strong interconnection between the NLRP3 sensor which stimulate adaptor protein ASC oligomerization and initiates inflammasome assembly^32^.

NLRP3 knock-down attenuated the up-regulation of NLRP3 and ASC, but had no significant effects on the expression of inflammasome effectors, such as caspase-1 and IL-1β^33^. Similar to the results obtained after gene expression analysis and caspase-1 and IL-1β production quantification, the expression of caspase-1 slightly increased in NLRP3 knock-down cells and maintained increased after PTX treatment suggesting the activation of caspase-1 dependent cell death (Fig. 8c). Pyroptosis initiation was also confirmed by IL-1β profile in PTX-treated cells before and after NLRP3 knock-down as suggested by some the morphological characteristics of pyroptosis, such as expansion of the cells. Altogether, these results indicate that NLRP3 knock-down can effectively inhibit the activation of NLRP3 inflammasome by inhibiting the expression of NLRP3 sensor, ASC, caspase-1 and IL-1β.

The quantification of green fluorescence revealed a statistically increased profile of NLRP3 after PTX-treatment compared to untreated control (*p* < 0.01) but significantly decreased compared to PTX-treated NLRP3 knock-down cells (*p* < 0.0001). Similar profile was obtained for ASC-associated red fluorescence indicating the up-regulation of ASC in contact with PTX (*p* < 0.01) and a significant difference of ASC expression in PTX-treated cells before and after NLRP3 knock-down (*p* < 0.001) (Fig. 8d). Statistically increased profile of red fluorescence associated with caspase-1 and IL-1β in MDA-MB-231 cells treated with PTX (*p* < 0.001 and *p* < 0.01 respectively) indicated the activation of pyroptosis cell death (Fig. 8e). On the other hand, no statistically significant but slightly decreased profile of both caspase-1 and IL-1β in contact with PTX before and after NLRP3 knock-down demonstrated the ability of PTX to stimulate tumor cell death.

## Discussion

TNBC is characterized by aggressive malignant transformation and progression with unpredictable evolution and poor response to classical treatment. Another important components of breast carcinogenesis are the pro-inflammatory microenvironment, inflammatory cells and mediators which determine the appearance of complications, such as genome instability and immune evasion, and the need to develop new therapies that target the inflammatory component associated with cancer^34^.

The NLRP3 inflammasome plays a central role in innate immunity and homeostasis. The activation of this complex determines caspase-1 maturation and activation of IL-1β and IL-18 which contribute to host-defense mechanism, inflammatory responses elaboration and breast cancer development^35^. Moreover, active caspase-1 determines GSDMD cleavage promoting pyroptotic cell death. Pyroptosis is associated with nuclear condensation, DNA fragmentation, cellular swelling and lysis, IL-1β and IL-18 release, all this contributing to an aggressive inflammatory response^36^. Abnormal expression of inflammasome components contributes to the pathogenesis of numerous inflammation-mediated diseases, including cancer^37^. Scientific literature supports the hypothesis according to which TNBC microenvironment is characterized by overexpression of NLRP3 inflammasome components^38^. Therefore, there is a great interest in the establishment of new therapeutic strategies (based on pharmacological inhibitors or gene silencing mechanism) capable to target the components or products of the NLRP3 inflammasome. In this context, the aim of the current study was to obtain an effective PTX-based anti-tumor strategy targeting NLRP3 inflammasome using both pharmaceutical modulation assisted by ATP and MCC950 and gene silencing protocol using NLRP3 specific siRNA.

First of all, the administration of PTX facilitated NLRP3 complex assembly and pyroptosis activation in MDA-MB-231 tumor cells. The obtained results indicated an increased expression of canonical components of inflammasome, such as NLRP3 sensor, ASC adaptor protein and caspase-1 effector after PTX treatment of tumor cells suggesting the activation of NLRP3 signaling pathway and implicitly caspase-1 dependent cell death initiation (pyroptosis). On the other hand, NOD2 belongs to NODosome subgroup of NLR family which is involved in immunosurveillance and is able to initiate an inflammatory response via NF-kB and mitogen-associated protein kinase (MAPK) signaling pathways^39^. The PTX-increased expression of NOD2 suggests its anti-tumor activity and capacity to significantly increase the chemosensitivity of MDA-MB-231 cells to PTX, being able to increase the pyroptosis rate of tumor cells after treatment. On the other hand, AIM2 is a cytoplasmic innate immune receptor that assembles inflammasomes, determining the release of IL-1β and IL-18 and pyroptosis^40^ and facilitates tumor cells proliferation. In vitro studies indicated the role of AIM2 as tumor suppressor and its capacity to inhibit breast cancer cell growth and proliferation via pyroptosis cell death^41^. After PTX treatment, the expression of AIM2 significantly increased compared to untreated control suggesting the initiation of cell death program and cancer cell proliferation suppression. IL-1β, IL-18 and GSDMD are executors of inflammasome activity due to caspase-1 capacity to cleave GSDMD and to release the N-terminal pore-forming domain and induce pyroptosis which facilitates the release of cell contents^42^. Moreover, GSDMD-derived pores facilitate water influx into cytosol determining cell swelling and lysis^43^. Another protein from gasdermin family is the tumor suppressor GSDME which is associated with apoptosis to pyroptosis conversion^44^. Recent studies indicated the role of GSDME in mediating the effectiveness of anti-tumor treatments and the adverse responses to drugs^45^. Also, low expression of GSMDE is associated with reduced sensitivity of Luminal A breast tumor cells to taxol derived drugs^45^ and the absence of alternative pathway of inflammasome activation via caspase-3^46^. Our results were in accordance with the literature suggesting the absence of GSDME-derived pores and low expression of caspase-3 before and after PTX-treatment, highlighting the relationship between GSDME and caspase-3^30,47^. Thus, the first part of our study strongly suggested the ability of PTX to support NLRP3 inflammasome signaling in TNBC context.

PTX-based therapies remain the standard of care for patients diagnosed with TNBC. Recent studies focused on the capacity of anti-tumor drugs to influence the immune response in the TME and observed that PTX possess immunostimulatory properties^48^. Other studies suggested the capacity of PTX to induce M1 macrophage polarization by TLR4/NF-kB signaling pathway stimulation, as indicated by the expression of pro-inflammatory markers, such as TNFα or IL-12^49^. Furthermore, PTX was also associated with the immunogenic cell death that triggers adaptive immunity through the release of DAMPs and activation of TLR4 signaling pathway^50^. Emerging evidence suggests the dual role of pyroptosis in tumorgenesis and the capacity of chemotherapeutic drugs, such as PTX, to induce pyroptosis in breast cancer models^51^. Pyroptosis or pro-inflammatory programmed cell death possesses morphological, biochemical and molecular particularities, such as cell swelling and plasma membrane rupture, that stimulate the recruitment of immune cells, the formation of an immunogenic TME and the activation of tumor immunity which implicitly promote tumor growth suppression^52^. Tumor progression is also orchestrated by cell to cell or cell to extracellular matrix adhesion molecules, such as integrins which mediate cell survival in the presence of apoptotic stimuli. The interaction between integrins and cytokine receptors promotes tumor metastasis and chemoresistance mechanisms^53^. Moreover, a recent study suggested that PTX treatment supported integrin profile modification in breast cancer cells, promoting the conformational changes in the breast TME^54^.

Therefore, the development of NLRP3 inflammasome and pyroptosis-derived therapeutic strategies using chemotherapy drugs or non-coding RNA molecules is necessary due to anti-apoptotic nature of tumor cells. The conducted study illustrated the effects of pharmacological or genetic regulation of NLRP3 inflammasome in order to facilitate the anti-tumor effects of PTX for TNBC eradication. In this regard, LPS and ATP were used for canonical NLRP3 inflammasome activation and pyroptosis induction, while MCC950 was used for specific inhibition of NLRP3 and IL-1β release, after which PTX was added to suppress the proliferation of tumor cells and programmed cell death initiation.

As described by Zha et al., ATP successfully induced inflammasome activation and pyroptosis in LPS-primed murine cells as indicated by cell membrane swelling and lysis and increased NLRP3, mature caspase-1 and active IL-1β levels^55^. As expected, our results illustrated that LPS and ATP determined statistically increased expression of inflammasome components, such as NLRP3, ASC, NOD2, as well as the effectors of this complex, such as caspase-1, IL-1 β, IL-18 and GSDMD, while GSDME low expression suggested the lack of treated tumor cells apoptosis.

MCC950 affects IL-1β secretion in a dose-dependent manner. In 2019, Coll et al. described the synthesis protocol, chemical structure and mechanism of action of MCC950 in both ex vivo and in vivo experiments. MCC950 is a small molecule inhibitor of the NLRP3 inflammasome that blocks the canonical, non-canonical and alternative mechanism of NLRP3 activation by interacting with the NLRP3 NACHT domain^22^. MCC950`s mechanism of action is based on the interaction with the Walker B motif of the NACHT domain which prevents the hydrolysis of ATP required for NLRP3 inflammasome activity^24^. Despite the promising properties, MCC950`s mechanism of action has not been completely understood. Recent studies focused on MCC950 investigation and the results indicated that the NLRP3 inhibitor did not inhibit mitochondrial respiration or reactive oxygen species production^56^ or calcium signaling^57^.

The researchers evaluated the MCC950 influence on NLRP3 inflammasome in LPS-primed and ATP-activated cells and the obtained results revealed that MCC950 reduced in a dose-dependent manner the activation of caspase-1, inhibited the release of IL-1β and blocked NLRP3-dependent pyroptosis initiation, but did not affect potassium efflux. Additionally, MCC950 capacity to inhibit the activation of AIM2 was studied and similar to our results, the experimental data suggested that MCC950 did not affect AIM2 activity. The final results revealed that MCC950 treatment altered NLRP3-induced ASC oligomerization and formation of ASC specks, which suggests that MCC950 can inhibit NLRP3 activation downstream of potassium efflux^57^.

The NLRP3 inflammasome performs a dual role in cancer depending on the TME and BC subtype^58^. Deng et al., suggested that the NLRP3 inflammasome activation stimulated by ROS generation, promoted the metastatic cascade in breast tumor cells^59^, while Xiong et al. proposed the anti-tumor role of inflammasome in immunogenic therapies^60^. Another study indicated that the inhibition of the NLRP3 inflammasome stimulated the recruitment and activation of Natural Killer cells in invasive breast cancer model, independently of IL-1β and IL-18 signaling^61^. Considering the complexity of NLRP3 inflammasome signaling in BC development and progression, advanced studies are needed to identify the effects of NLRP3 inhibition on breast TME. In line to our findings, PTX effects on NLRP3 inflammasome has been confirmed in preexistent studies. For example, Zheng et al. investigated the consequences of PTX activity on NLRP3 inflammasome activation on LPS-primed cells in response to ATP stimulation. The results revealed that PTX triggered ASC specks and NLRP3 complex formation and pyroptosis initiation as suggested by high levels of caspase-1, IL-1β and GSDMD^62^.

Therefore, this is the first study reporting the effects of LPS, ATP, MCC950 and PTX combination on NLRP3 inflammasome in TNBC conditions. The obtained results highlighted both the MCC950-specific inhibition and PTX-enhanced NLRP3 inflammasome activation, but the effective collaboration between MCC950 and PTX was displayed by inflammasome-related markers expression analysis. Further, NLRP3 knock-down was performed using a specific siRNA molecule in order to demonstrate the crucial role of NLRP3 inflammasome in TNBC progression and the consequences of NLRP3 suppression on the chemotherapeutic treatment.

As expected NLRP3, ASC and NOD2 gene expression decreased after incubation with NLRP3 siRNA and maintained decreased even after PTX treatment suggesting the successful suppression of the NLRP3 inflammasome oligomerization mechanism. On the other hand, AIM2 expression significantly increased after NLRP3 knock-down and PTX treatment indicating its role as a tumor suppressor and as an intracellular DNA receptor which detects damaged DNA molecules^63^. As described by Liu et al., breast cancer associated AIM2 up-regulation can decrease the expression of anti-apoptotic B-cell lymphoma-extra large (Bcl-xL) and increase the expression of pro-apoptotic Bcl-2-associated protein x (Bax) determining the inhibition of breast cancer cell growth^64^. The expression of inflammasome effectors (caspase-1, IL-1β, GSDMD) increased after NLRP3 knock-down and PTX treatment suggesting the initiation of programmed cell death. Recent studies revealed that only NLRP3 silencing suppressed breast tumor cells proliferation and induced cell death, which represents an advantage for obtaining a favorable post-chemotherapeutic response^38,65^.

Numerous in vitro studies suggested the dynamic role of NLRP3 inflammasome as a signaling platform for inflammatory responses elaboration which contributes to cancer progression by providing a favorable environment. In vitro studies present abundant advantages, such as reduced cost, low variability, high stability and reproducibility, but at the same time fail to reproduce in vivo cell behavior and are restrained to anticipate in vivo biological processes. In this study, we highlighted the PTX mechanism of action in TNBC compared to normal conditions and the initial results suggested the activation of NLRP3 inflammasome and implicitly of an inflammatory process. In the second part of this study, we proposed a possible anti-tumor strategy that involved NLRP3 inflammasome modulation using both pharmacological and molecular inhibitors followed by the classical administration of the anti-tumor agent PTX. The obtained results suggested that inhibition of NLRP3 inflammasome and associated inflammatory processes stimulated the cytotoxic effects of PTX on TNBC cells, reducing at the same time the risk of side effects after chemotherapy.

The publication of scientific studies that illustrates the comparison between in vitro and in vivo systems are limited due to the biological complexity and interconnected physiological pathways. In this context, our study provides an improved understanding of the complex interconnection between NLRP3 inflammasome activity, pyroptosis cell death and TNBC progression, opening new opportunities for the development of efficient NLRP3 inflammasome targeted therapies in TNBC pathology. However, the execution and optimization of in vivo protocols are imperative for the evaluation of possible anti-tumor therapies. One of the approaches that could be utilized to achieve this aim could be in silico study, which can reduce the use of animal models in pre-clinical studies.

## Conclusion

In this study, we evaluated the effects of NLRP3 inflammasome modulation on the anti-tumor activity of PTX in TNBC context, highlighting the interconnection between inflammatory mechanisms and tumor progression. The results indicated the pro-inflammatory effects of PTX as revealed by increased expression of NLRP3 inflammasome components and pyroptosis effectors in MDA-MB-231 cells, as compared with MCF-12A cells. Pharmacological inhibition of NLRP3 inflammasome using MCC950, followed by PTX administration decreased the pro-inflammatory processes associated with anti-tumor treatment as indicated by the expression of NLRP3, caspase-1, IL-1β and IL-18, compared with PTX treatment. Furthermore, siRNA transfection results suggested the beneficial effects of NLRP3 knock-down on PTX treatment as indicated by the decreased expression of pro-inflammatory markers, compared to PTX treatment only. All of these findings may contribute to a better understanding of the impact of NLRP3 inflammasome activity on TNBC pathogenesis, opening new opportunities for targeted therapy. The molecular mechanism of NLRP3 inflammasome in TNBC progression is very elaborate and requires advanced in vivo studies before performing preclinical studies.

## Methods

### Materials

The MCF-12A (CRL-10317) and MDA-MB-231 (HTB-26) cell lines were obtained from ATCC, Manassas, USA, and tested for mycoplasma contamination. Dulbecco’s Modified Eagle’s Medium (DMEM, D2902), Dulbecco’s Modified Eagle′s Medium/Nutrient Mixture F-12 Ham (DMEM-F12, D2906), antibiotic antimycotic solution (A5955), phosphate-buffered saline (PBS) powder (P4417), Cholera toxin from *Vibrio cholerae* supplement (C8052), human epidermal growth factor (hEGF) supplement (E9644), hydrocortisone supplement (H0888), bovine serum albumine (BSA, A7906), paraformaldehyde (PFA) solution (47608), saponin (47036) and Hoechst 33,258 solution (62249) for fluorescence staining nuclei, lipopolysaccharides (LPS) from *Escherichia coli* (l4391) and TRIzol reagent (15596018) were purchased from Sigma-Aldrich, Steinheim, Germany. Fetal bovine serum (FBS, 10270-106) was purchased from ThermoFisher Scientific, Waltham, MA, USA. Adenosine triphosphate (ATP, tlrl-atpl) and MCC950 (inh-mcc) were purchased from Invivogen, Toulouse, France. Paclitaxel powder (P3456), goat anti-mouse AlexaFluor 488 (A11029) and goat anti-rabbit AlexaFluor 546 (A11010) secondary antibodies were purchased from Invitrogen, Waltham, MA, USA. Caspase-Glo 1 Inflammasome Assay (G9951) and Lumit Human IL-1β Immunoassay (W6010) were purchased from Promega, Madison, WI, USA and iScript cDNA Synthesis kit (170–8891) was purchased from BioRad, Hercules, CA, USA. Forget-Me-Not Evagreen qPCR Master mix (31045) was purchased from Biotium, Fremont, CA, USA. Caspase-1 (2225 S), ASC (13833), cleaved IL-1β (83186 S) and cleaved caspase-3 (9664) antibodies were purchased from Cell Signaling Technology, Inc., Danvers, MA, USA. NLRP3 (sc-518123) antibody, NLRP3 siRNA (sc-45469), control siRNA (Fluorescein Conjugate)-A (sc-36869), siRNA transfection reagent (sc-29528) and siRNA transfection medium (sc-36868) were purchased from SantaCruz Biotechnology, Inc., USA.

### Cell culture and subcultivation

MCF-12A are non-tumorigenic epithelial cells isolated from the mammary gland, while MDA-MB-231 are tumor cells derived from adenocarcinoma, with metastatic properties, poorly differentiated and highly invasive (ER negative, PR negative, HER2 negative), being significant in vitro models for the study of anti-TNBC targeted therapies^66^. MCF-12A cells were cultured in DMEM-F12 media supplemented with 10% FBS, 1% antibiotic-antimycotic solution, 100 ng/mL Cholera toxin from *Vibrio cholerae* supplement, 20 ng/mL hEGF supplement and 500 ng/mL hydrocortisone supplement, while cells from MDA-MB-231 cell line were cultured in DMEM media supplemented with 10% FBS and 1% antibiotic-antimycotic solution. The culture media was changed once every two-three days until reaching 70–80% confluence, after which the cells were subcultured through enzymatic treatment. The cells were incubated under standard culture conditions, at 37°C and 5% CO_2_, in a humidified atmosphere.

### In vitro experimental model

#### PTX treatment

MCF-12A and MDA-MB-231 were seeded at 2,5 × 10^4^ cells/cm^2^ and at 2 × 10^4^ cells/cm^2^ respectively, and incubated for 24 h under standard conditions to allow cell adhesion. The normal and tumor cells were then treated with 100 nM PTX for 24 h and gene and protein expression levels of inflammasome specific markers were determined to evaluate the effects of PTX on NLRP3 inflammasome activity.

#### NLRP3 complex modulation

NLRP3 inflammasome modulation was performed in order to investigate its involvement in TNBC progression. MCF-12A and MDA-MB-231 were seeded at 2,5 × 10^4^ cells/cm^2^ and at 2 × 10^4^ cells/cm^2^ respectively, and incubated for 24 h under standard conditions to allow cell adhesion. The cells were treated with 1 µg/mL LPS solution for 4 h in order to obtain the priming signal needed for NLRP3 activation. The activation of the NLRP3 inflammasome was carried out by incubating the cells with 5 mM ATP for 1 h. Afterwards, the normal and tumor cells were incubated for 24 h with 10 µM MCC950 inhibitor or 100 nM PTX. In some experimental conditions, a combination of 10 µM MCC950 and 100 nM PTX was used to observe the effects of inflammasome inhibition and PTX cytotoxicity on TNBC evolution. Gene expression study, Caspase-Glo 1 Inflammasome Assay and Lumit Human IL-1β Immunoassay were carried out at 24 h after pharmacologic inhibition of NLRP3 inflammasome in LPS and ATP-treated cells.

#### NLRP3 siRNA transfection

To reduce endogenous NLRP3 expression, MDA-MB-231 cells were transfected with NLRP3 specific siRNA according to the manufacturer`s protocol. MDA-MB-231 cells were seeded at 2 × 10^4^ cells/cm^2^ density in antibiotic-free normal growth medium supplemented with 10% FBS and incubated for 24 h in standard conditions to allow cell adhesion after which NLRP3 siRNA transfection reagent was added. 1 µg siRNA duplex diluted in 100 µL siRNA Transfection Medium and 6 µL siRNA transfection reagent diluted in 100 µL siRNA Transfection Medium were mixed and incubated 30 min at room temperature. Afterwards, the transfection mixture was diluted with 800 µL siRNA Transfection Medium and added over the washed cells. The cells were incubated with the siRNA transfection reagent mixture for 7 h in standard condition. After 7 h, normal growth medium containing 20% FBS and 2% antibiotic-antimycotic solution was added over the cells without removing the transfection mixture. After 24 h, the transfection mixture was replaced with growth medium supplemented with 10% FBS and 1% antibiotic-antimycotic solution and the cells were incubated for an additional 72 h. The transfection efficiency was evaluated by fluorescence microscopy using siRNA (Fluorescein Conjugate)-A and qRT-PCR analysis of NLRP3 gene expression. After the successful knock-down of NLRP3, the cells were treated with 100 nM PTX for another 24 h and Caspase-Glo 1 Inflammasome Assay, Lumit Human IL-1β Immunoassay, gene and protein expression levels were evaluated using the corresponding techniques.

### Gene expression-quantitative real-time PCR

The control and treated cells underwent an evaluation of the expression levels of genes involved in the activity of the NLRP3 inflammasome using the quantitative Real-Time PCR (qRT-PCR) technique. Total RNA of each condition was isolated using the classic method based on the use of TRIzol reagent. The concentration of the obtained total RNA was measured using NanoDrop 8000 spectrophotometer (Thermo Fisher Scientific, USA) and the complementary DNA synthesis was realized employing iScript cDNA synthesis kit and the Veriti 96-Well Thermal Cycler (Applied Biosystems), according to the manufacturer`s protocol. qRT-PCR reactions were performed using ViiA 7 Real-Time PCR system (Applied Biosystems, USA), Forget-Me-Not EvaGreen qPCR Master Mix and specific primers` sequences (Table 1), according to the manufacturer`s protocol. The samples were processed in triplicate, and glycerol aldehyde phosphate dehydrogenase (GAPDH) was tested as a reference gene.

**Table 1:** Primers` sequences.

Gene		Nucleotide sequence
GAPDH	F	GTCTCCTCTGACTTCAACAGCC
	R	ACCACCCTGTTGCTGTAGCCAA
NLRP3	F	GCACGTGTTTCGAATCCCAC
	R	CCTGCTGGCTCCGGTGCTCC
ASC	F	GCGCTGGAGAACCTGACCGC
	R	CTCCTGCAGGCCCATGTCGC
NOD2	F	GCACTGATGCTGGCAAAGAACG
	R	CTTCAGTCCTTCTGCGAGAGAAC
AIM2	F	CTGCACCAAAAGTCTCTCCTCATG
	R	GGCTGAGTTTGAAGCGTGTTGAT
Caspase-1	F	GCCTGTTCCTGTGATGTGGAG
	R	TGCCCACAGACATTCATACAGTTTC
IL-1β	F	TCTGTACCTGTCCTGCGTGT
	R	ACTGGGCAGACTCAAATTCC
IL-18	F	CCAGCCTGACCAACA
	R	CCACAACCTCTACCTCC
GSDMD	F	GTGTGTCAACCTGTCTATCAAGG
	R	CATGGCATCGTAGAAGTGGAAG
GSDME	F	TGCCTACGGTGTCATTGAGTT
	R	TCTGGCATGTCTATGAATGCAAA

### Caspase-1 activity-Caspase-Glo 1 Inflammasome Assay

To assess the activity of NLRP3 inflammasome, the level of caspase-1 released in the culture media of the treated cells was measured using Caspase-Glo 1 Inflammasome Assay. Caspase-1 activity was monitored and selectively determined employing a homogeneous and bioluminescent method based on Z-WEHD substrate cleavage by caspase-1 resulting in the luciferase reaction and light production. The assay provides Z-WEHD-aminoluciferin and MG-132 inhibitor which allow the fast detection of caspase-1 activity, but also YVAD-CHO reagent which is a caspase-1 selective inhibitor used to distinguish caspase-1 inflammasome activity from that of other caspases. The assay was performed following manufacturer’s indications and the luminescent signal was measured after 60 min of incubation, using FlexStation 3 spectrophotometer (Molecular Devices, San Jose, CA, USA).

### IL-1β release-Lumit Human IL-1β Immunoassay

IL-1β released from treated cells into culture media as a consequence of inflammasome activation and caspase-1 maturation was measured using Lumit Human IL-1β Immunoassay. The principle of this assay is based on a luminescent structural complementation system capable of recognizing and binding to IL-1β, generating a bright luminescent signal directly proportional to the amount of analyte present in the sample. A standard curve of known concentrations (40 ng/mL − 22 pg/mL range) of human IL-1β was prepared and used to quantify the IL-1β levels from each samples. The culture media collected from each experimental condition was mixed with 5X anti-hIL-1β antibody mixture in 1:1 ratio and incubated for 60 min. Following incubation, the plate was allowed to equilibrate to room temperature and the samples were incubated with 5X Detection Reagent B for 5 min. The generated luminescence was measured using FlexStation 3 spectrophotometer.

### Protein expression-immunostaining coupled with confocal microscopy

Protein expression levels of the specific NLRP3 inflammasome components were investigated by immunostaining coupled with confocal microscopy. The treated and control cells were fixed with a 4% PFA solution for 1 h and permeabilized with 0.1% saponin solution for 20 min, at 4°C. Afterwards, the samples were incubated overnight at 4°C with NLRP3 mouse monoclonal antibody diluted 1:100, ASC rabbit monoclonal antibody diluted 1:100, cleaved IL-1β rabbit monoclonal antibody diluted 1:100, caspase-1 rabbit polyclonal antibody diluted 1:200 and cleaved caspase-3 rabbit monoclonal antibody diluted 1:800. Subsequently, the samples were incubated for 1 h at room temperature, in dark conditions, with anti-mouse AlexaFluor 488 and anti-rabbit AlexaFluor 546 secondary antibodies diluted 1:500. Cell nuclei were stained with Hoechst solution. The images revealing inflammasome-associated protein expression levels before and after treatment with PTX were visualized and analyzed using to Nikon AX R Eclipse Ti2-E Confocal Microscope system during a workshop organized by ELTA’90 MR as Nikon representatives in Romania. The images revealing NLRP3, ASC, caspase-1 and IL-1β protein expression levels in NLRP3 knock-down cells were visualized and analyzed using Nikon A1/A1R Confocal Laser Microscope System confocal microscope and corresponding software. Quantification of the fluorescence levels for the selected markers was performed using ImageJ software.

### Statistical analysis

All experiments conducted were carried out in triplicate (*n* = 3) and the results obtained were expressed as mean ± standard deviation using the GraphPad Prism 6.0 software (GraphPad Software Inc., USA). Statistical significance was assessed using the same software, through the One-way ANOVA method and the Bonferroni algorithm, considering a statistical difference for *p* < 0.05.

## Data Availability

All data are available in the main text. The datasets analyzed during the current study are available from the corresponding author upon reasonable request.
